# Relative reduction of plasmacytoid dendritic cells with shift in TH_1_ to TH_2_ response in HIV-1 infected patients as compared to high risk and healthy north Indians

**DOI:** 10.1186/1471-2334-12-S1-O20

**Published:** 2012-05-04

**Authors:** Omkar Chaudhary, Manju Bala, Jasbir Singh, Anjali Hazarika, Rajesh Kumar, Kalpana Luthra

**Affiliations:** 1 All India Institute of Medical Sciences, New Delhi 110029, India; 2 Regional STD Teaching Training & Research Centre, Safdarjang Hospital, New Delhi, India; 3 Kurukshetra University, Kurukshetra 136119, India; 4 Society for Promotion of Youth and Masses Center, Vasant Kunj, Delhi, India

## Background

Dendritic cells (DCs) are professional antigen presenting cells and play a central role in both innate and adaptive immunity. A decrease in one or both subsets of DC has been reported in HIV-1 infected patients from different populations. The status of DC subsets in subjects at high risk for HIV-1 such as Injecting Drug Users (IDUs) has not been reported so far.

## Methods

Blood samples from 15 healthy individuals, 15 IDU and 15 HIV-1 positive patients were collected and informed consent was obtained. Plasmacytoid and myeloid DCs were accessed by four-color flow cytometry. The plasma level cytokines and HIV-1 viral load were determined.

## Results

We observed a significant decrease in the total DCs and pDCs population in HIV-1 infected patients (%DCs p = 0.0132, %pDCs p = 0.0281) and IDUs (%DCs p = 0.006, %pDCs (p>0.0001) as compared to healthy individuals. The plasma levels of IFN-γ was significantly lower while level of IL-10 was significantly higher in HIV-1 infected patients as compared to IDUs (p = 0.0062, for IFN- γ and p = 0.0071 for IL-10) and healthy subjects (p = 0.004 for IFN- γ and p = 0.0068 for IL-10).

## Conclusions

This is the first study to characterize the dendritic cells subpopulations in IDUs who are at high risk for HIV-1 infection. Further longitudinal studies on the status of dendritic cell subpopulations and their correlation with the cytokine profile will enable the elucidation of the precise role of dendritic cells in HIV-1 infection.

